# A macular horseshoe tear following posterior vitreous detachment and longstanding branch retinal vein occlusion

**DOI:** 10.3205/oc000264

**Published:** 2025-12-10

**Authors:** Konstantina Manoli, Jared Ching

**Affiliations:** 1 Bristol Eye Hospital, Bristol, United Kingdom; 2 King’s College Hospital, London, United Kingdom; 3 Department of Engineering Science, University of Oxford, United Kingdom

**Keywords:** horseshoe macular tear, idiopathic macular tear, longstanding branch retinal vein occlusion, posterior vitreous detachment

## Abstract

**Objective:**

We present a case of a macular horseshoe tear that demonstrated signs of progression over time that was therefore deemed sight threatening. There are no reports that provide a definitive means to treat such a rare pathology.

**Methods:**

Spectral domain optical coherence tomography (SD-OCT) scans were used to monitor subretinal fluid at each follow up. Once confirmation of the accumulation of subretinal fluid was made, vitrectomy surgery including amputation of the horseshoe tear flap, short acting gas tamponade and face down positioning were utilized.

**Results and conclusion:**

Macular horseshoe tears can be treated with a vitrectomy approach akin to retinal detachment repair without the need for retinopexy.

## Introduction

Macular horseshoe tears are rare and have been associated with previous blunt trauma and recurrent ischemic retinal vein occlusions. The mechanism for the development of a macular tear is unclear, however it has been suggested that chronic macular oedema after a branch retinal vein occlusion (BRVO) [1] and an ischemic macula [2] are predisposing factors for a macular tear when a posterior vitreous detachment (PVD) occurs. In the case of blunt trauma, the forces exerted to the globe lead to expansion and contraction, transmitting forces through the retina that can result in a macular tear. However, a number of reports of macular horseshoe tears have not identified an underlying cause and are therefore described as idiopathic.

Herein, we describe the outcome of a case of a progressive unilateral macular horseshoe tear in a 76-year-old female patient treated with a vitreoretinal surgical approach.

## Case description

A 76-year-old Caucasian female was referred to the eye casualty of our hospital in February 2023 by her optometrist due to new onset of floaters from her left eye that started two weeks ago, after she bent over and lifted a heavy flowerpot while gardening.

Her past ophthalmic history included bilateral cataract surgery 3 years ago and a non-ischemic supero-temporal BRVO in her left eye 20 years ago for which a conservative approach was taken.

Snellen visual acuity was 6/7.5 (LogMAR 0.10) in the right eye and 6/12 (LogMAR 0.30) in the left. The intraocular pressure was 14 mmHg bilaterally.

**Figure 1:**
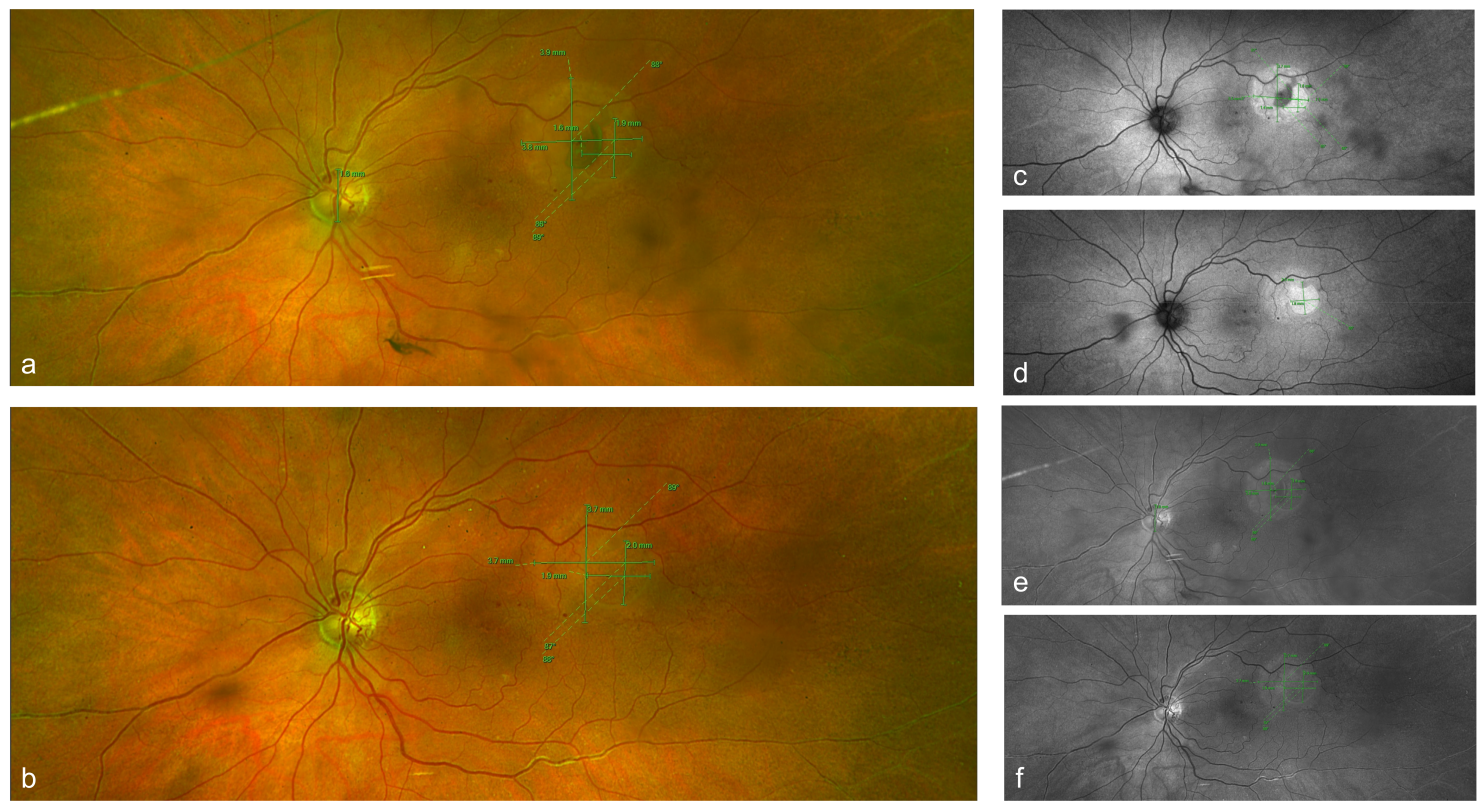
Cropped optos widefield images (left panels: pseudocolour, top right panels: autofluorescence, and bottom right panels: red channel) of the left eye posterior pole and horseshoe macular tear. a) pseudocolour image of the posterior pole of the left eye, where the macular horseshoe tear is seen supero-temporal from the macula, at the day of presentation. Subretinal fluid size 3.9x3.8 mm, retinal tear size 1.9x1.6 mm, b) postoperative outcome, retinal hole size 1.9 x2 mm, no subretinal fluid. c) and d pre- and post-operation autofluorescent images. e) and f) pre-and post-operation red channel images.

**Figure 2:**
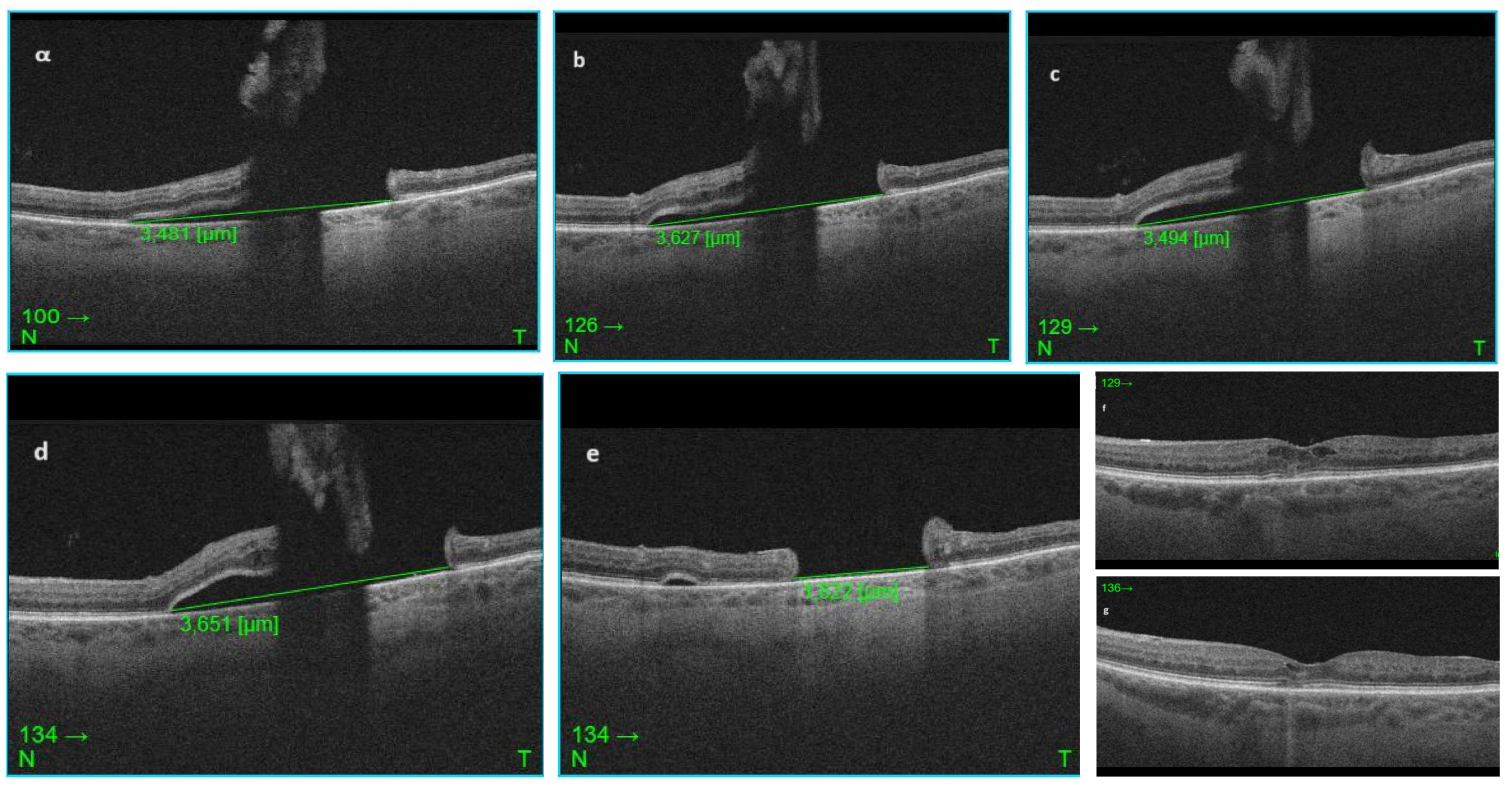
SD-OCT Images of the macular horseshoe tear through the same position demonstrating the change in the subretinal fluid at each follow up. The size of the fluid has been manually measured using the calipers of the Topcon software. a) shows the B-scan of the day of diagnosis with SRF size 3.481 µm, b) shows the scan at one week follow up, base of the tear measured 3.627 µm, c) at two weeks follow up, the SRF is reduced, base of the tear measured 3.494 µm but the retinal flap appears more raised and d) shows the one month follow up scan, where there is an increase in the amount of SRF size 3.651 µm and definite progression. e) post-surgical macular defect measuring 1.822 µm, flat, with no evidence of subretinal fluid or residual vitreous traction, f) and g) show pre- and post-surgical macular oedema.

Slit lamp examination of the left eye revealed quiet pseudophakia, with an intact posterior chamber intraocular lens sited in the bag, clear media, positive Shaffer’s sign, and PVD present. A macular horseshoe tear of one disc diameter was found in the supero-temporal region of the macula of the left eye with surrounding subretinal fluid measuring almost 3-disc diameters (3.9x3.8 mm) on ultrawide field imaging (Optos, Dunfermline, UK) (Figure 1a (Fig. 1)). Peripheral retinal examination revealed no degenerations, tears or retinal detachment. No retinal vascular changes were noted apart from optic disc collateral vessels from the known supero-temporal non-ischemic BRVO. Mild cystoid macular oedema (CST 320) and intraretinal cysts in the nasal detached retina of the macular horseshoe tear were present in the spectral-domain optical coherence tomography (SD-OCT) (Triton^TM^ plus DRIOCT, Topcon Healthcare) with no evidence of vitreous traction (Figure 2a, f (Fig. 2)).

The patient was offered three approaches including observation, laser retinopexy and vitrectomy surgery. She preferred to be observed in the first instance and only consider treatment if there was subjective or objective evidence of progression. For this reason, measurements of the subretinal fluid diameters using corresponding SD-OCT B-scan images were taken at each follow up over a month and compared (Figure 2 (Fig. 2)).

Written consent was provided by the patient for the publication of her data and images in a scientific journal according to the ethics committee of the hospital.

At one week follow up, the patient’s vision was 6/12 (LogMAR 0.30) in the affected eye, the intraocular pressure 14 mmHg and her symptoms stable. No progression was evident on either fundoscopy or OCT imaging, where the base of the retinal tear measured 3.627 µm (Figure 2b (Fig. 2)).

At two weeks follow up, the left eye visual acuity was 6/15 (LogMAR 0.40) and the intraocular pressure 14 mmHg. No progression was recorded, and even a mild improvement in the SRF with the base of the tear measuring 3.494 µm was observed (Figure 2c (Fig. 2)).

One month after the original examination the patient noticed no change of the paracentral scotoma, her vision from the left eye was 6/12 (LogMAR 0.30), the intraocular pressure 12 mmHg, but it was noted that the subretinal fluid associated with the horseshoe macular tear had progressed (Figure 2d (Fig. 2): base of the tear measured 3.651 µm).

Given the evidence on SD-OCT imaging that there was progressive subretinal fluid accumulation, the patient agreed that intervention was necessary to avert the risk of retinal detachment that could affect the macula. Retinal laser retinopexy was discussed and ruled out as this would be associated with central visual field loss and a decision was made to pursue vitrectomy surgery without any retinopexy. 

A 25-gauge 3 port pars plana vitrectomy (Constellation^®^ Vision System, Alcon, Geneva, Switzerland) was performed. A thorough core and peripheral vitrectomy was performed with amputation of the flap of the horseshoe macular tear using the vitrector with active aspiration and a cut rate of 7,500 cuts per minute. No retinopexy was performed and vitreous cavity tamponade with 20% SF6 was used. Sutureless closure of the sclerotomies was performed with subconjunctival betamethasone and cefuroxime to complete the procedure. The patient was positioned for one hour face down immediately postoperatively. No further posturing was advised.

The patient was monitored for 6 months following surgery, serial SD-OCT images demonstrated that the pathology was stable, with a retinal defect size of 1.822 µm (Figure 2e (Fig. 2)) and no evidence of new tears or breaks. Her final Snellen visual acuity 6 months after surgery was 6/6 (LogMAR 1) in the right eye (Nd: Yag laser was performed in this eye as well) and 6/15 (LogMAR 0.40) in the left, intraocular pressure 13 mmHg in the right and 14 mmHg in the left eye.

The patient was discharged from the vitreoretinal clinic thereafter and has not attended the Emergency Eye Department for over a year.

## Conclusions

Few cases of macular horseshoe tears have been published. Herein, we present an extra-foveal horseshoe macular tear following a long-standing non-ischemic BRVO treated with vitrectomy and gas alone. 

The presence of a previous non-ischemic supero-temporal BRVO, with the macular horseshoe tear occurring in the same region, suggests a possible association secondary to cystoid oedema or retinal ischemia. However, the preceding BRVO was at least 20 years ago, and the location of the macular horseshoe tear was near the termination of the supero-temporal arcade, suggesting that posterior vitreous perivascular adhesion may have contributed to the formation of the retinal tear upon the occurrence of a PVD. However, the possibility that this was an idiopathic pathology cannot be completely excluded. Karim-Zade et al. [3] reported a case of a macular tear following recurrent BRVOs in the same eye and retinal ischemia, whereas in our case there was no clinical evidence of retinal ischemia, however interestingly, mild macular oedema was present. The acute onset of floaters occurred after the patient lifted a heavy object, along with the progressive subretinal fluid accumulation at the macular horseshoe tear, suggests that the PVD may have been the precipitating event. As such, we rationalized treating this as a peripheral horseshoe tear, i.e., removing the vitreous traction, although none was evident, by amputating the horseshoe tear flap and avoiding any retinopexy given the posterior location of the break.

Numerous techniques to treat large macular tears and holes have been published, including internal limiting membrane (ILM) peel and inverted ILM flap, autotransplantation of the ILM, amniotic membrane graft, and autologous anterior capsule graft. To our knowledge, there is no record of treatment of a macular tear more than 1.5 mm, let alone a successful closure of one. 

ILM peel is performed for idiopathic macular hole surgery to release abnormal vitreoretinal interface forces at the macula. However, in our case the underlying pathology differs; the macular horseshoe tear was likely caused by a PVD rather than an abnormal vitreoretinal interface disorder. Therefore, we rationalized that treating this horseshoe tear as macular hole, as previously described, was not warranted. Further, given that the horseshoe tear was extrafoveal, there would be limited benefits in terms of paracentral visual field gains or prevention of metamorphopsia, given the difference in underlying pathology.

As such, a vitrectomy, amputation of the flap of the tear and tamponade with a short acting gas (SF_6_) without any retinopexy was performed. This contrasts with others who treat similar horseshoe tears as macular holes, using an inverted ILM flap technique. 

Rishi et al. [4] compared the outcomes of idiopathic macular hole surgery in eyes with randomized SF_6_, C_2_F_6_ and C_3_F_8_ gas tamponade and concluded that there is no statistically significant benefit of one over the other. Shorter acting gases impart an earlier recovery and quicker visual rehabilitation and as such, we elected to use SF_6_.

Finally, our case differs due to the position of the horseshoe macular tear and the surgical approach. The horseshoe tear was sited in the extrafoveal region and likely associated to a PVD later leading to symptomatic progression.

Treatment with a vitrectomy, horseshoe tear flap amputation and short acting gas tamponade, similar to a retinal detachment repair, barr retinopexy, differentiates our case from those previously published and demonstrates a straightforward vitrectomy approach can be utilized in such clinical presentations (Table 1 (Tab. 1)).

## Notes

### Informed consent

Written consent was signed by the patient. The study/case report was performed in the Bristol Eye Hospital.

### Competing interests

The authors declare that they have no competing interests.

## Figures and Tables

**Table 1:**
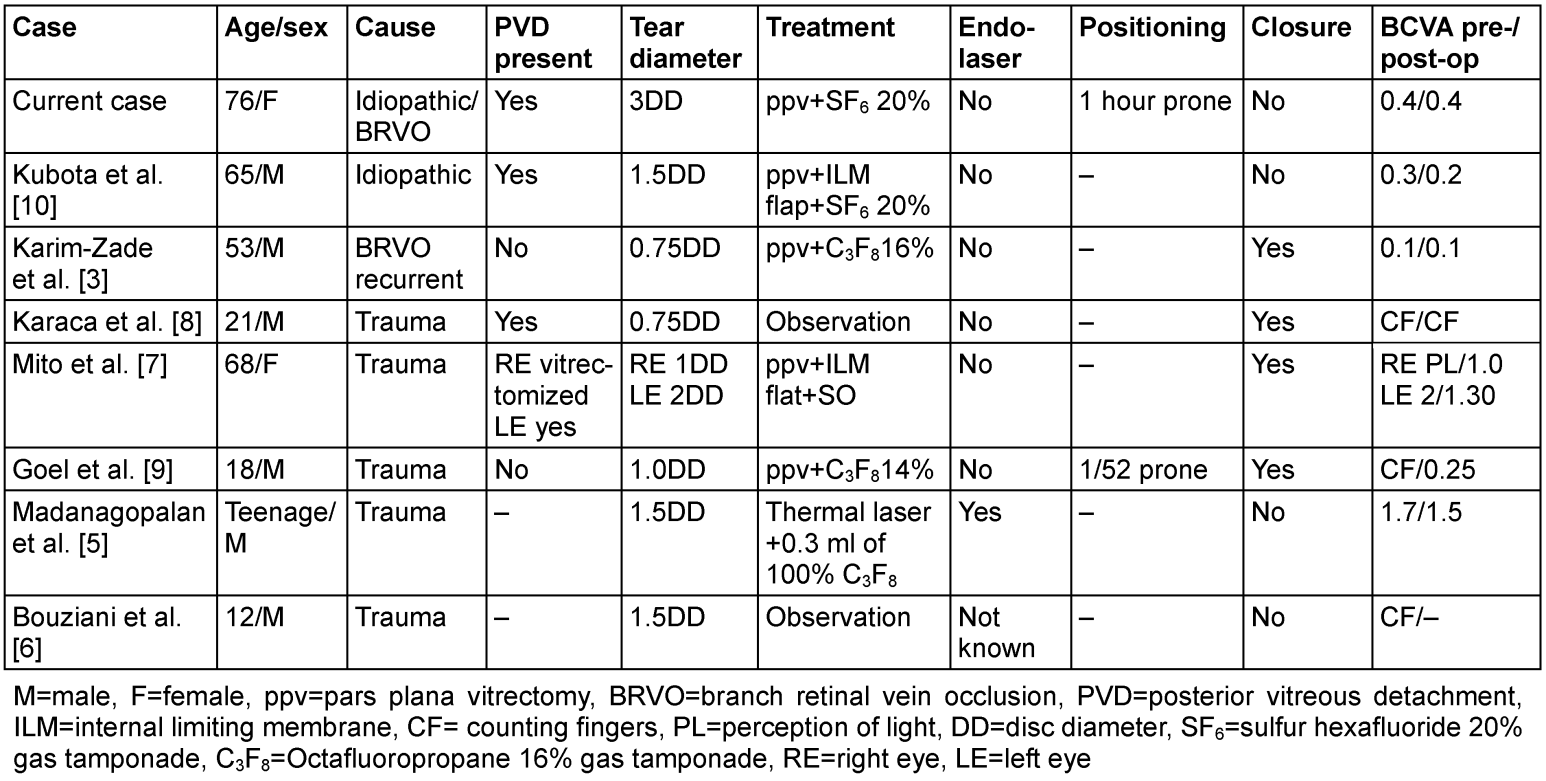
Comparison of reported macular tears, with underlying causes, including trauma, following a BRVO, or for no clear reason (idiopathic). Different operating approaches have been used to treat those including observation, vitrectomy with ILM flap, pneumatic retinopexy or vitrectomy alone. This table demonstrates that ours and Kubota et al.’s [10] case are the only cases with possible idiopathic macular horseshoe tears. Our case has the largest tear dimensions reported to this day.
